# A comparative study on Ca content and distribution in two Gesneriaceae species reveals distinctive mechanisms to cope with high rhizospheric soluble calcium

**DOI:** 10.3389/fpls.2014.00647

**Published:** 2014-11-20

**Authors:** Wenlong Li, Falun Xu, Shixuan Chen, Zhennan Zhang, Yan Zhao, Yukuan Jin, Meijing Li, Yan Zhu, Yongxiu Liu, Yi Yang, Xin Deng

**Affiliations:** ^1^Key Laboratory of Plant Resources and Beijing Botanical Garden, Institute of Botany, The Chinese Academy of SciencesBeijing, China; ^2^Key Laboratory of Plant Molecular Physiology, Institute of Botany, Chinese Academy of SciencesBeijing, China; ^3^College of Life Sciences, University of Chinese Academy of SciencesBeijing, China; ^4^Key Laboratory of Bio-Resource and Eco-Environment, Ministry of Education, College of Life Sciences, Sichuan UniversityChengdu, China

**Keywords:** *Boea hygrometrica*, *Lysionotus pauciflorus*, karst plant, heat shock protein, chaperon, high Ca stress, photosynthesis and the environment

## Abstract

Excessive Ca is toxic to plants thus significantly affects plant growth and species distribution in Ca-rich karst areas. To understand how plants survive high Ca soil, laboratory experiments were established to compare the physiological responses and internal Ca distribution in organ, tissue, cell, and intracellular levels under different Ca levels for *Lysionotus pauciflorus* and *Boea hygrometrica*, two karst habitant Gesneriaceae species in Southwest China. In the controlled condition, *L. pauciflorus* could survive as high as 200 mM rhizospheric soluble Ca, attributed to a series of physiological responses and preferential storage that limited Ca accumulation in chloroplasts of palisade cells. In contrast, *B. hygrometrica* could survive only 20 mM rhizospheric soluble Ca, but accumulated a high level of internal Ca in both palisade and spongy cells without disturbance on photosynthetic activity. By phenotype screening of transgenic plants expressing high Ca-inducible genes from *B. hygrometrica*, the expression of *BhDNAJC2* in *A. thaliana* was found to enhance plant growth and photosynthesis under high soluble Ca stress. *BhDNAJC2* encodes a recently reported heat shock protein (HSP) 40 family DnaJ-domain protein. The Ca-resistant phenotype of BhDNAJC2 highlights the important role of chaperone-mediated protein quality control in Ca tolerance in *B. hygrometrica*. Taken together, our results revealed that distinctive mechanisms were employed in the two Gesneriaceae karst habitants to cope with a high Ca environment.

## Introduction

Crops and vegetables with high Ca contents are potential sources of dietary Ca for human use (Dayod et al., 2010). Fertilization and biofortification via transgenic manipulation have been used to increase calcium in food crops. However, the progress has been limited, essentially because that excessive intracellular calcium causes toxicity to plants. Though Ca is an essential macronutrient for plants with key structural and signaling roles (Dayod et al., 2010; Gilliham et al., 2011), excessive intracellular Ca can precipitate with phosphates, prevent seed germination, disturb photosynthesis, reduce growth rate and form tiny yellowish or gold spots of fruits (Chan et al., 2003; White and Broadley, 2003). Ca is mainly obtained from the soil solution through the root system in the form of Ca^2+^, and transported in the xylem from root to shoot driven by transpiration pull and soil Ca^2+^ concentration (White, 2001; Conn and Gilliham, 2010). Ca becomes relatively immobile once having been transported into cells (Malone et al., 2002). Thus, external Ca supply and transpiration have major impacts on the amount of plant internal Ca. Many plants decrease transpiration when external Ca elevates to limit Ca uptake (Silva et al., 1996; Tang et al., 2007). Therefore, finding a way to control plants Ca and accumulation without disturbing normal growth and survival under high Ca condition is a key step for generation of plants with high Ca contents.

Karst terrain is famous for Ca-rich soil, which has been one of the major factors that limit the species and production of crops and vegetables that could be cultivated. However, it is an undeniable natural advantage for cultivation of crops and vegetables with high Ca contents. Moreover, the native habitant plants in karst area have provided valuable resources for understanding how plants survive high Ca. Our previous survey on the plants in karst mountainous area in Southwest China have revealed a number of plants that always kept low internal Ca contents in aboveground tissues regardless of the high calcium level in soil (High-Ca type), that always accumulated high internal Ca contents even in the relatively low calcium soil (Low-Ca type), and that could varied their Ca contents in accordance with soil Ca contents (Variable-Ca type) (Ji et al., 2009). Understanding how these plants absorb, transport, store and tolerate high level internal Ca in Ca-rich soil will pave the way for generation of crops and vegetables that contain high Ca contents, and that could be cultivated in karst area. Such effort will eventually benefit the development of agriculture, ecosystem and economics in the vast karst regions, which accounts for about 15% of the world's land area or about 2.2 million km^2^, and is home for around 1 billion people (17% of the world's population).

*Lysionotus pauciflorus* and *Boea hygrometrica* are two species in Gesneriaceae family, both native habitants in karst rocky areas in Southwest China (Wang et al., 2005). *L. pauciflorus* is a small shrub with procumbent small stolons or rhizome, while *B. hygrometrica* is a kind of herb that has been well characterized as a resurrection plant, whererby its vegetative organs can tolerate extreme dehydration (Mitra et al., 2013). Several genes and proteins have been identified to play roles in desiccation tolerance in *B. hygrometrica* and other Gesneriaceae resurrection plants (Jiang et al., 2007; Liu et al., 2009; Zhao et al., 2014; Chen et al., in press), yet no information at the molecular level has been obtained for understanding how these plants cope with high Ca soil so far. In this study, a survey on the rhizospheric Ca contents of karst plants in Southwest China revealed that *L. pauciflorus* exhibited the highest amount of soluble Ca in the rhizospheric soil among all tested species. We then performed laboratory experiments to compare the threshold of the external soluble Ca that *L. pauciflorus* and *B. hygrometrica* could survive, the physiological responses and internal Ca distribution in different organs, tissues and cell types under different rhizospheric calcium application. The possible mechanisms that plants employed to survive high calcium in *L. pauciflorus* and *B. hygrometrica* were discussed.

## Materials and methods

### Plant materials and treatments

Plants of 10 species, namely Eriophorum comosum, Oplismenus undulatifolius, Cyrtomium fortunei, Pieris multifida, Selaginella moellendorffii, Corchoropsis tomentosa, Eupatorium adenophorum, Boea hygrometrica, Lysionotus pauciflorus, Paraboea rufescens, were collected from typical karst regions in Libo (107°44′ E, 25°15′ N), Luodian (106°51′ E, 25°33′ N), Huajiang (105°36′ E, 25°41′ N), and Puding (105°45′ E, 26°14′ N) in Guizhou province of China. Young plants of L. pauciflorus and B. hygrometrica were grown in quartz sand with irrigation of 1/2 strength Hoagland nutrient solution (Hoagland and Arnon, 1950) in a greenhouse. After 15 days, different concentrations of Ca(NO_3_)_2_ were applied with 1/2 strength Hoagland nutrient solution for 7 days. For L. pauciflorus and B. hygrometrica, photographic and physiological records were only documented for five concentrations, 2.5 (control), 20, 60, 100, and 200 mmol·L^−1^, because all leaves of L. pauciflorus dropped when plants were treated with 350–900 mmol·L^−1^ Ca(NO_3_)_2_ and no leaf was available for physiological measurement.

### Determination of Ca contents in soil and in plant

Rhizosphere soil samples were prepared by collecting soil surrounding the roots of individual plants from the native habitats. Soil samples were oven-dried, and passed through a 2-mm sieve. Then 5 g samples were soaked in 50 mL deionized water (for soluble Ca) or 1 mol·L^−1^ NH_4_COOH (for exchangeable Ca) and shaken for 30 min. The mixture was filtered quickly. 1 mL 10% La(NO_3_)_3_ was then added to 10 mL filtrate.

Aboveground and underground parts of plants were washed with deionized water and blotted dry. The samples were then dried at 80°C for 48 h in an oven. Dried samples were ground, and 0.2 g of the powder was digested in H_2_SO_4_:H_2_O_2_ (5:3, v/v).

Ca content was determined by Atomic Absorption Spectrophotometer (5100 PC, the Perkin-Elmer Corporation, USA) (Mizuno and Minami, 1980).

### Net photosynthetic rate, stomatal conductance, water content, and transpiration rate

Net photosynthetic rate, stomatal conductance, and transpiration rate were determined using LI-6400XT portable photosynthesis system (LI-6400XT, LI-COR, USA) (Lü et al., 2010). Net photosynthetic rate was computed by measuring the rate of change of CO_2_ with time with a leaf enclosed in a relatively large chamber as follow: Net photosynthetic rate = F × [Cr−Cs × ((1000−Wr)/(1000−Ws))]/100S. F represents flow rate to the sample chamber (μmol·s^−1^). Cr and Cs represent CO_2_ concentration of reference chamber and sample chamber (μmol CO_2_ mol^−1^), respectively. Wr and Ws represent H_2_O concentration of reference and sample chamber (mmol H_2_O mol^−1^), respectively. S represents leaf area. Plant samples were oven-dried for 48 h and water content was calculated as: Water content = (Fresh weight – Dry weight)/Fresh weight × 100%.

### SEM X-ray microanalysis

Plant leaves were frozen in liquid N and fractured with pre-cooled razor blades and then freeze-dried using a vacuum freeze-drier at −80°C for 3 days, to ensure that the water in biological materials can be removed with optimized structure preservation, and cellular mobile macromolecules and mobile ions can be stabilized without redistribution (Edelmann, 2002). Low temperature was kept to avoid possible Ca redistribution during sample preparation, as suggested by Edelmann (2002). Dried samples were mounted on aluminum stubs, and coated with gold. Elemental analysis was performed using an energy-dispersive X-ray microanalysis system (EMAX 350, Horiba, Japan) fitted to a HITACHI S-4800 scanning electron microscope (SEM) (Hitachi, Japan). The acceleration voltage used was 20 kV. X-ray spectra were collected for 180 s each. At least three replicates of X-ray line scans were obtained for each sample and three independent analyses were performed. The signal of endogenous magnesium was used as a reference to evaluate the effects of variation of surface topography between the different selected cells on the efficiency of SEM X-ray rate counting (Olmos and Hellin, 1998; Peng et al., 2004; Fernandez-Garcia et al., 2009).

### ^45^Ca feeding and autoradiograghy

*L. pauciflorus* and *B. hygrometrica* seedlings grown in unstressed condition were used for ^45^Ca-feeding experiments. Radioactive ^45^Ca was added proportionally into 1/2 strength Hoagland nutrient solution containing different concentrations of Ca to final activities as follows: 0.2 MBq ml^−1^ (2.5 mM), 2 MBq ml^−1^ (20 mM), and 20 MBqml^−1^ (200 mM). Plants were transferred to quartz sand and irrigated with the nutrition solution containing relative ^45^Ca content for 7 days. Eight plants were treated for each condition. Four plants were used for autoradiography of ^45^Ca in a dark room, and the rest for radioactivity measurement using a liquid scintillation counter (XH-6925, China) (Busse and Palta, 2006; Kerton et al., 2009).

### Fluo-3 AM staining

Transverse sections of the leaves were prepared using two razor blades in MES buffer (25 mM, pH 6) to minimize wound effect. Pluronic F-127 (20% in dimethylsulfoxide, Invitrogen, USA) was mixed (1:1) with the stock solutions of Fluo-3 AM (11.05 mM stock in dimethylsulfoxide, Invitrogen, USA), before addition of MES buffer. The sections were stained at 4°C for 2 h in the dark with 22.1 μM Fluo-3 AM mixed with 0.04% Pluronic F-127 in MES buffer (25 mM, pH 6). Low temperature was kept to avoid cleavage of acetoxymethyl group from fluo-3 AM by extracellular esterases that activate the probe, as suggested and Zhang et al. (1998). After staining, the sections were washed in MES buffer for 2 h at 20°C in the dark. The fluorescence signal was observed using a Zeiss LSM 510 META confocal laser scanning microscope (Zeiss Microsystems, Germany) using excitation at 488 nm. The emission was detected at 515–580 nm for Fluo-3 AM (Zhang et al., 1998; Albrechtova et al., 2003; Zienkiewicz et al., 2011).

### Examination of stress tolerance in transgenic *Arabidopsis*

Transgenic *Arabidopsis thaliana* plants overexpressing *B. hygrometrica* genes *BhC2DP1, BhDNAJC2*, and *BhOAR1* were obtained previously (Zhang et al., 2012; Zhao et al., 2014; Chen et al., in press). Seeds of T3 generation of three homozygous lines of each gene were surface sterilized and germinated on 1/2 Murashige and Skoog (MS) agar plates in parallel with wild-type controls at 22°C with a 16 h light/8 h dark cycle. Three day-old seedlings were transferred to 1/2 MS agar plates containing 60 mM CaCl_2_ for high-Ca stress phenotyping. The position of the seed lots were kept consistent in each treatment in one set of experiments but arranged randomly in different sets of repetitions. At least three independent experiments were conducted with three plates and 6 seedlings per line per plate were assayed. Photographs were taken, primary root length and physiological parameters were determined after 10 days of growth. In all cases, only wild-type seed batches that were generated in parallel with the transgenic seed lines were used for controls. Photochemical efficiency (Fv/Fm) and the extent of electrolyte leakage were measured as described previously (Jiang et al., 2007; Chen et al., in press).

### RNA isolation and real-time PCR

Total RNA was isolated using the trizol method with TRIzol Reagent (Takara, D9108B). After digestion with DNase I, it was reverse transcribed into cDNA with Oligo(dT)18 primer using SuperScript III reverse transcriptase, and used as template for PCR amplification. 18S rRNA was used as internal references. Real-time PCRs were performed in a Mastercycler® ep Realplex apparatus (Eppendorf, Hamburg, Germany) with SYBR® Green Real-time PCR Master Mix (TOYOBO, Japan). The relative expression levels were determined using 2^−ΔΔCt^ method (Livak and Schmittgen, 2001). The expression level of each transcript was measured in three independent biological samples with three technical replicates.

### Statistical analysis

All data were subjected to a One-Way ANOVA followed by DUNCAN (alpha = 0.05) test using SAS9.2, and expressed as means ± standard deviation. Each data was obtained from at least three samples.

## Results

### *L. pauciflorus* tolerate high rhizospheric soluble Ca

**Figure 1:**
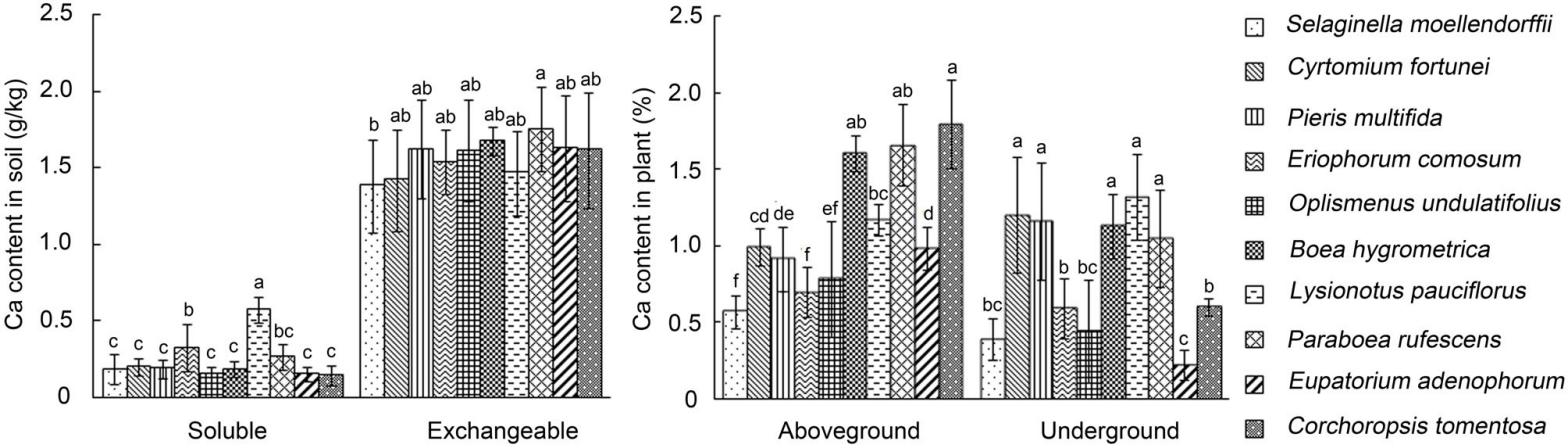
**Rhizospheric and internal Ca contents of plants collected from karst areas in Southwest China**. Soluble and exchangeable Ca contents in soil and in different (aboveground and underground) parts of plants were measured using atomic absorption spectrophotometry. Data presented as means ± SD (*n* = 3). The different lowercase letters on the bars indicate significantly different means (*P* < 0.05).

The calcium content in calcareous soils in Southwest China karst areas is 2–3 times more than that in red and yellow soil (Cao et al., 2003). To investigate the capacity of karst plants to survive high Ca environment, plants and rhizospheric soils of 10 species that were commonly found in Southwest China karst areas, including 3 ferns (*C. fortunei, P. multifida* and *S. moellendorffii*), 2 grasses (*E. comosum* and *O. undulatifolius*), and 5 dicots (*C. tomentosa, E. adenophorum* and three species of Gesneriaceae, e.g., *B. hygrometrica, L. pauciflorus*, and *P. rufescens*), were collected from their natural habitats and determined for total Ca contents. The results showed that the levels of exchangeable Ca content in the rhizospheric soils were similar among these plants (1382–1752 mg/kg), which were 2–5 fold higher than that in loamy sand (300 mg/kg), silt loam (650 mg/kg), and sand loam (700 mg/kg) (Simmons and Kelling, 1987; Gunter and Palta, 2008). The highest soluble Ca content of the rhizosphere soils was found with *L. pauciflorus* (nearly 600 mg/kg), which was significantly higher than the others (143–320 mg/kg) (Figure 1). On the other side, Ca content in shoots and roots of *L. pauciflorus* were similar to *B. hygrometrica* (1.6%) and *P. rufescens* (1.2%), but higher than some of the other species tested. These data suggest that *L. pauciflorus* exhibits extraordinary ability to cope with high soluble Ca in the rhizosphere.

### The ranges of external Ca concentrations that *L. pauciflorus* and *B. hygrometrica* can survive

**Figure 2:**
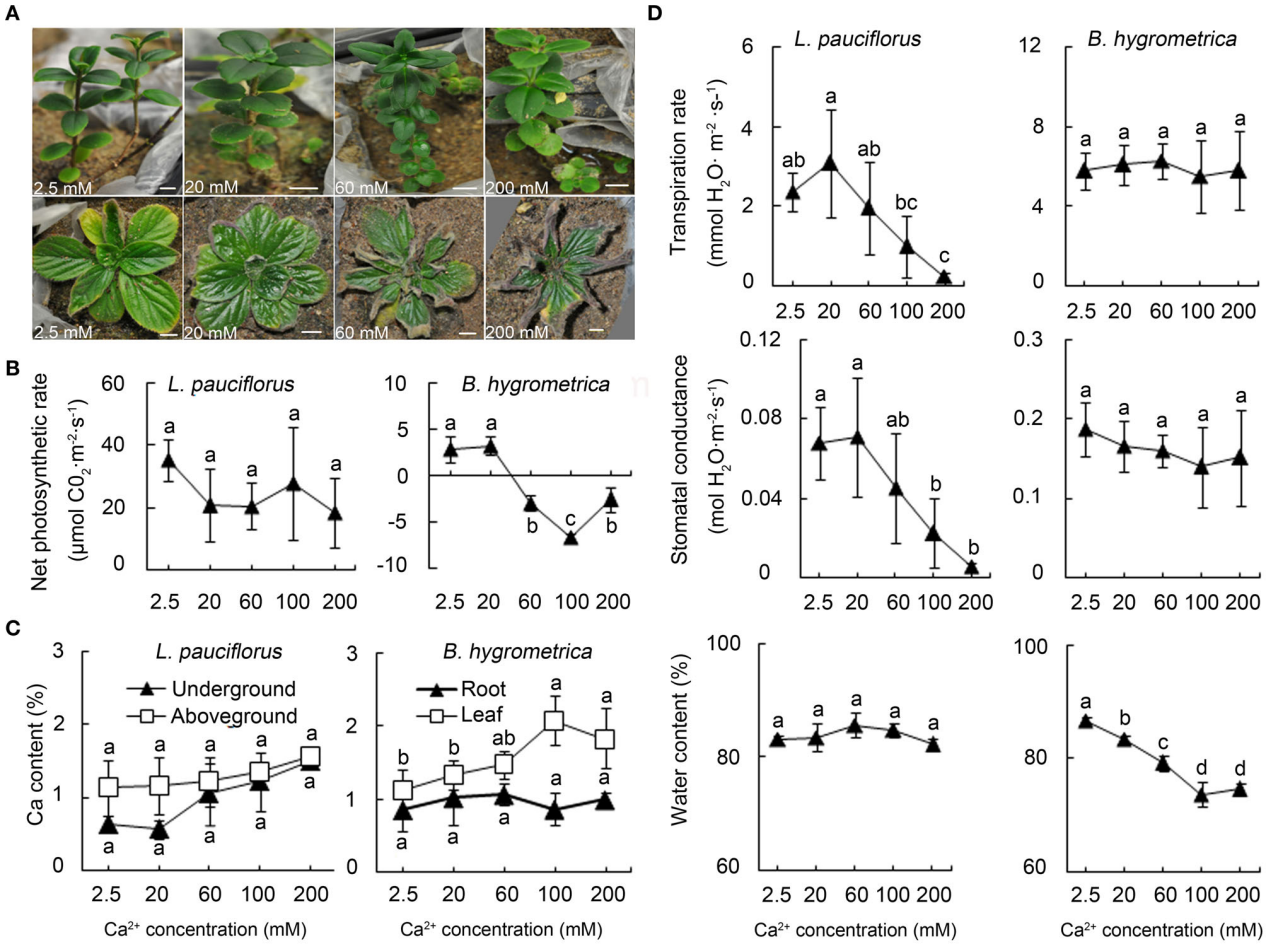
**Growth and physiological parameters of *L. pauciflorus* and *B. hygrometrica* under different Ca conditions**. Plant seedlings were grown in sand and irrigated with ½ Hoagland nutrient solutions containing different amounts of Ca for 7 days. **(A)** The growth of *L. pauciflorus* (upper panel) and *B. hygrometrica* (lower panel) under different Ca conditions. Scale bar = 1 cm. **(B)** Net photosynthetic rates of leaves of *L. pauciflorus* and *B. hygrometrica* plants. **(C)** Ca contents in different parts of *L. pauciflorus* and *B. hygrometrica* plants. **(D)** Transpiration rate, stomatal conductance, and water content of *L. pauciflorus* and *B. hygrometrica* plants. The mean ± SD is shown (*n* = 3). The different lowercase letters on the bars indicate significantly different means (*P* < 0.05).

The soluble Ca content of the rhizosphere soil of *L. pauciflorus* and *B. hygrometrica* in the karst area was nearly 600 mg/kg (15 mmol/kg) and 200 mg/kg (5 mmol/kg), respectively. However, these values do not represent the threshold of high Ca stress that *L. pauciflorus* and *B. hygrometrica* could survive. To quantitatively define the tolerance and the threshold of external high Ca conditions for both plants, a laboratory experimental system was established to cultivate plants under high Ca treatments by adding 2.5–900 mM Ca(NO_3_)_2_ in sand. This system can effectively monitor rhizospheric soluble Ca content in greenhouse conditions. It was shown that *L. pauciflorus* plants grew well under 2.5–200 mM external soluble Ca and no toxic symptoms were observed at least within a week (Figure 2A); however, leaves became yellowish and fell down under 350–900 mM Ca(NO_3_)_2_. In contrast, *B. hygrometrica* displayed obvious toxic symptoms such as curved leaves when 60 and 200 mM Ca were applied (Figure 2A). *L. pauciflorus* could maintain active but slightly decreased photosynthesis activities under 20–200 mM external Ca conditions. In comparison, *B. hygrometrica* could maintain a regular level of photosynthesis under moderate Ca stress (20 mM Ca), but dropped sharply under higher Ca treatments (Figure 2B). According to plant growth and photosynthetic activities, the highest rhizospheric soluble Ca threshold is 200 mM for *L. pauciflorus* plants, and 20 mM for *B. hygrometrica* under our cultivation conditions.

To determine whether the sensitivity to the above described treatments is due to high Ca rather than high NO^−^_3_ and high osmotic pressure, plants were also treated with 200 mM (for *L. pauciflorus*) or 20 mM (for *B. hygrometrica*) CaCl_2_ and Mg(NO_3_)_2_, respectively. Results showed that after 7 days, *L. pauciflorus* and *B. hygrometrica* plants under Ca(NO_3_)_2_ and CaCl_2_ treatments grew normally, displaying similar levels of net photosynthetic rates, water contents, and osmolalities as plants under control condition (Figure S1). In contrast, symptoms of leaf chlorosis and falling (*L. pauciflorus*) or curling (*B. hygrometrica*) were observed for plants under the treatment of Mg(NO_3_)_2_ (Figures S1A,B). Net photosynthetic rates and relative water contents were not affected in *L. pauciflorus* but significantly dropped in *B. hygrometric* plants under Mg(NO_3_)_2_ treatment (Figure S1C). The osmolalities were 500 mmol/kg for all these 200 mM salt solutions and 100 mmol/kg for 20 mM salt solutions (data not shown), therefore the phenotypes observed were not due to high osmolar pressure of the applied salt solutions. Mg(NO_3_)_2_ treatments led to higher levels of osmolalities in plants (Figure S1C), compared to Ca(NO3)_2_ and CaCl_2_ treatments, further supporting that the Ca resistance of *L. pauciflorus* and *B. hygrometrica* is specific to Ca, rather than another cation, or merely changes in water availability and osmotic pressure caused by the application of these salt solutions.

### The accumulation of Ca in aboveground and underground parts of plants after high Ca treatment

The effects of external high Ca on the plant internal Ca content were monitored by measuring the Ca contents in aboveground and underground parts of plants under different Ca levels. For *L. pauciflorus*, Ca contents were slowly increased from 1.1% to 1.6% in the aboveground part, and from 0.6 to 1.5% in the underground part, when plants were treated with 2.5–200 mM (Figure 2C). In contrast, root Ca content was stable at about 0.9–1% in *B. hygrometrica* when external Ca was 2.5–200 mM, whereas leaf Ca content gradually climbed from 1.1 to 2.1% when external Ca was increased from 20 to 100 mM and dropped to 1.8% when 200 mM Ca was applied (Figure 2C). Compared to the plants summarized by Dayod et al. (2010), which contained 0.13–460 mg Ca per 100 g dry weight, *L. pauciflorus* and *B. hygrometrica* plants possess 2.4–8461 fold higher internal Ca content, even under low Ca condition (2.5 mM). Furthermore, these data revealed that underground organs could serve as a Ca sink to reduce the load to leaves in *L. pauciflorus* when external Ca increases, whereas the roots of *B. hygrometrica* could not.

### Transpiration, stomatal conductance, and water content in plants under external high Ca condition

It had been demonstrated that transpiration rate was one of the major factors that drive the absorption and root-to-shoot transport of Ca in the xylem (Conn and Gilliham, 2010). Therefore, we examined the changes of transpiration rate, stomatal conductance and water content in plants grown under high external Ca. Data have shown that both transpiration and stomatal conductance were kept stable in 20 mM treated *L. pauciflorus* plants, but decreased markedly with the increase of external Ca from 60 to 200 mM (Figure 2D). In agreement, water contents remained at the stable levels in *L. pauciflorus* plants under all treatment. The decrease of transpiration may help to reduce Ca absorption and upwards transport in *L. pauciflorus* under high Ca condition. In comparison, no fluctuation of transpiration rate and stomatal conductance in *B. hygrometrica* was observed, which was supported by the drop of water content when plants were treated with 2.5–200 mM Ca (Figure 2D). These data implied that external Ca application affected plant water status, transpiration and stomatal closure in opposite manners in the two Gesneriaceae species. It is worth highlighting that *L. pauciflorus* appears to have a much lower stomatal conductance and transpiration rate anyway even under low Ca addition, suggesting that it is generally more suited to maintenance of water status.

### Ca uptake in *L. pauciflorus* and *B. hygrometrica* under high Ca conditions

**Figure 3:**
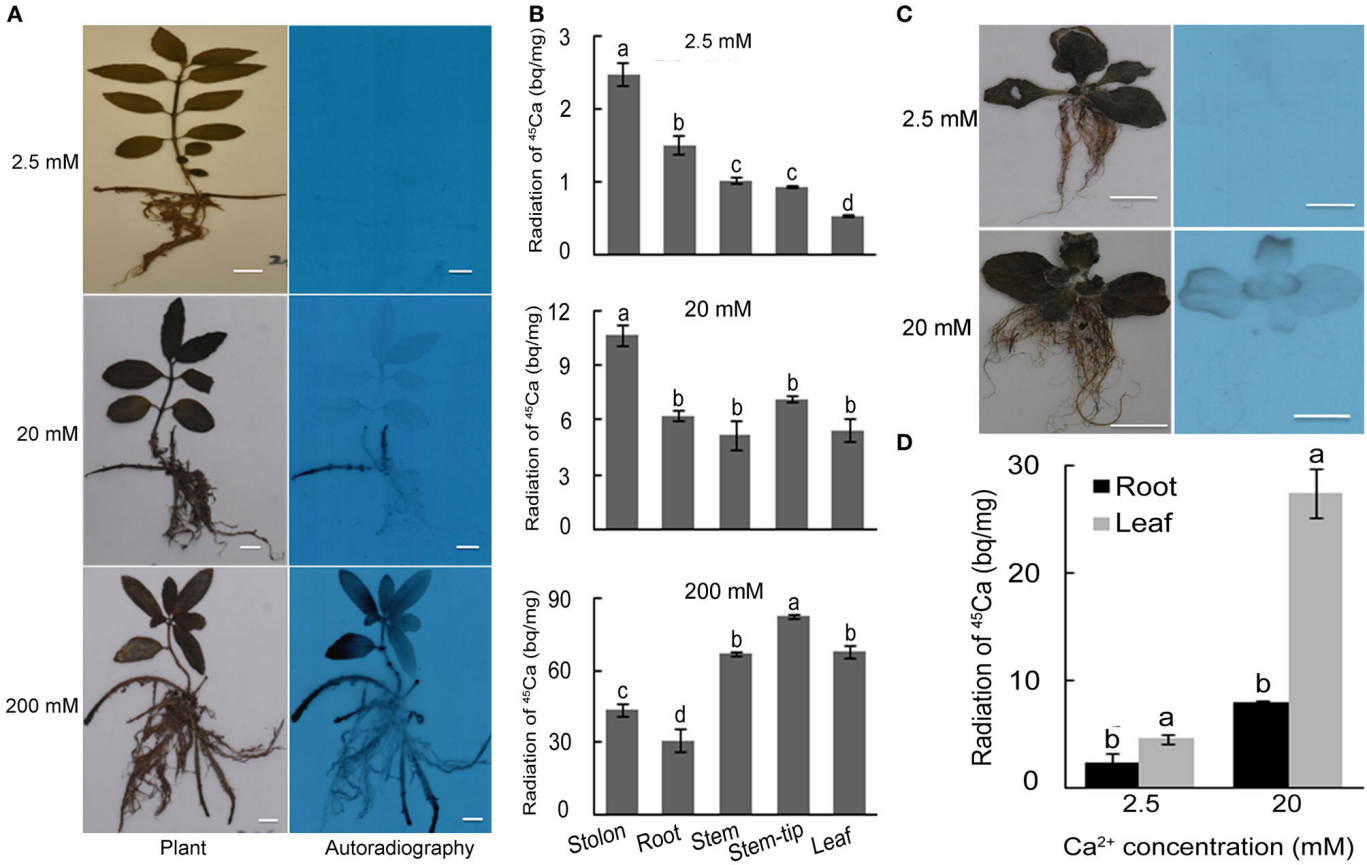
**^45^Ca autoradiography and scintillation counting of *L. pauciflorus* and *B. hygrometrica* plants after Ca treatments for 7 days. (A)** Autoradiography of *L. pauciflorus* plants treated with 2.5, 20, 200 mM Ca. Scale bar = 1 cm. **(B)** Radioactivity of ^45^Ca in different organs of *L. paucifloru*s. **(C)** Autoradiography of *B. hygrometrica* treated with 2.5, 20 mM Ca. Scale bar = 1 cm. **(D)** Autoradiography and radioactivity of ^45^Ca in different organs of *B. hygrometrica*. Mean values ± SD are shown (*n* = 3). The different lowercase letters on the bars indicate significantly different means (*P* < 0.05).

To detect Ca uptake and movement after the application of high Ca, seedlings of the two species were fed with ^45^Ca for 7 days in the cultivation condition described above. Autoradiography revealed only faint signals in stolons of plants grown under 2.5 mM Ca, intensive signals in stolons of plants grown under moderate Ca treatment (20 mM), and massive signals in leaves, stems, stolons and roots of plants grown under high Ca treatment (200 mM) (Figure 3A). The quantification of the radioactive intensity further confirmed that the ^45^Ca radioactivity in stolons was higher than that in all the other organs under 2.5–20 mM Ca conditions, indicating that stolons serve as a major underground Ca sink under moderate Ca conditions. The radioactivity in leaves and stems were higher than that in stolon and root, and a gradual decrease of ^45^Ca strength from root to stem tip and leaf was observed in plants grown under 200 mM Ca treatment (Figure 3B). This suggested that a large amount of Ca moved into leaves via stem when 200 mM Ca was applied in rhizosphere.

To the contrary, in *B. hygrometrica*, autoradiography revealed very faint signals in leaves of the plants grown under 2.5 mM Ca treatment, and strong signals in the edges of leaves but no signal in roots of plants under 20 mM Ca condition (Figure 3C). Quantification revealed that the Ca radioactive strength in leaves of 20 mM Ca-treated *B. hygrometrica* was 6 fold of that of 2.5 mM Ca-treated plants, and the Ca radioactive signals in roots of 20 mM Ca-treated plants were 3 fold of that of 2.5 mM Ca-treated plants (Figure 3D). The radioactive intensity in leaves of 20 mM Ca-treated *B. hygrometrica* plants were much higher than that in leaves of 20 mM Ca-treated *L. pauciflorus* plants, whereas the radioactive intensities in roots were similar between 20 mM Ca-treated plants of *L. pauciflorus* and *B. hygrometrica*. These results indicated that a leaf is the main organ in *B. hygrometrica* for Ca accumulation under moderate Ca conditions.

### Ca distribution in different cell types of leaves of plants under high calcium

**Figure 4:**
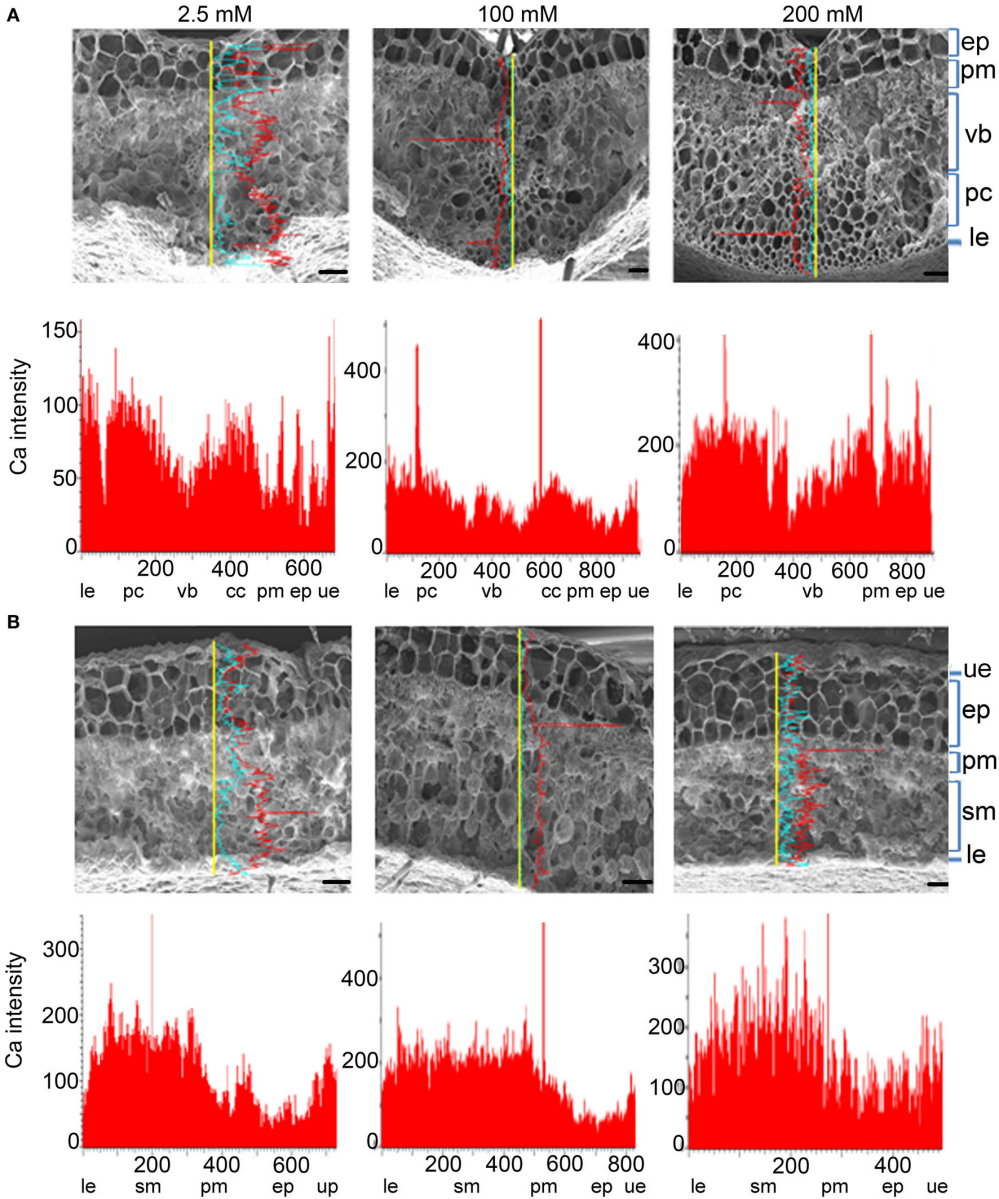
**Ca accumulation and distribution in the vein and non-vein parts of *L. pauciflorus* leaves. (A)** Vein. **(B)** Non-vein. Ca content was detected by SEM X-ray line profiles. Plants were treated with 2.5, 100, 200 mM Ca concentrations respectively for 7 days. Yellow line, the scanning line. Red curve, Ca intensity counts. Blue curve, Mg intensity counts. Y-axis, Ca intensity counts; X-axis, position on line scan (μm). Scale bar = 100 μm. ue, upper epidermis; pm, palisade mesophyll; cc, collenchyme; vb, vascular bundle; pc, parenchyma; ep, epithelial cells; sm, spongy mesophyll; le, lower epidermis.

*L. pauciflorus* leaves contain an amphicribral vascular bundle in the midrib (Figure 4A), a thick upper epidermis structure that is composed of one layer of parenchyma and two or three layers of large epithelial cells, a typical mesophyll structure that is composed of several layers of compact thin and small palisade cells and incompact middle-sized round spongy cells, and a hairless thin layer of lower epidermis cells (Figure 4B). As shown by SEM X-ray line profiles, a valley of Ca signal was detected in the vascular bundle in midribs in plants under all treatments, indicating the effective unloading to mesophyll cells (Figure 4A). The Ca intensities in spongy cells were always 2–3 fold more than that in palisade cells and upper epidermis in non-vein sections of leaves in all treatments (Figure 4B), indicating the spongy cells were main Ca storage locations in leaves.

**Figure 5:**
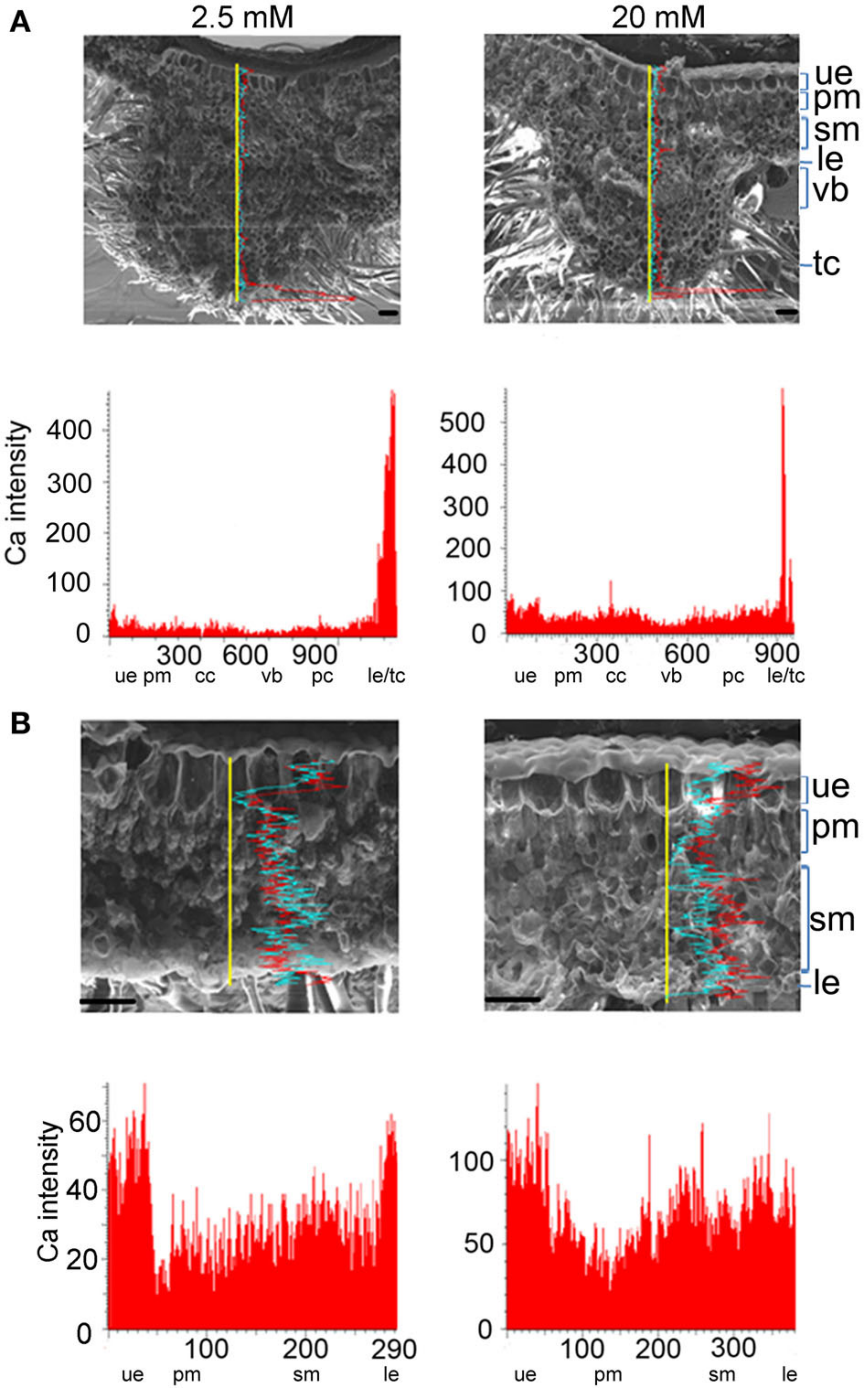
**Ca accumulation and distribution in the vein and non-vein parts of *B. hygrometrica* leaves. (A)** Vein. **(B)** Non-vein. Ca content was detected by SEM X-ray line profiles. Plants were treated with 2.5, 20 mM Ca concentrations respectively for 7 days. Yellow line, the scanning line. Red curve, Ca intensity counts. Blue curve, Mg intensity counts. Y-axis, Ca intensity counts; X-axis, position on line scan (μm). Scale bar = 100 μm. ue, upper epidermis; cc, collenchyme; pm, palisade mesophyll; sm, spongy mesophyll; le, lower epidermis; cc, collenchyme; vb, vascular bundle; pc, parenchyma; tc, trichome.

SEM X-ray imaging confirmed our previous observation of the leaves of *B. hygrometrica* by TEM (Wang et al., 2009) and line profiles revealed that Ca contents in all cell types were significantly increased with the rise of external Ca concentration (Figure 5). Among all cell layers in both the vein and non-vein sections, Ca intensities were high in the upper and lower epidermis and relatively low in mesophyll cells under 2.5–20 mM external Ca treatment (Figures 5A,B). This indicated that the epidermis and probably the hairs on it were the main sinks of Ca and mesophyll cells accumulated Ca when external Ca increased. The Ca distribution pattern was distinct from Mg distribution in both *L. pauciflorus* and *B. hygrometrica* leaves (Figures 4, 5), indicating the specific Ca accumulation pattern under high Ca conditions.

### Free Ca distribution in leaves of *L. pauciflorus* and *B. hygrometrica*

**Figure 6:**
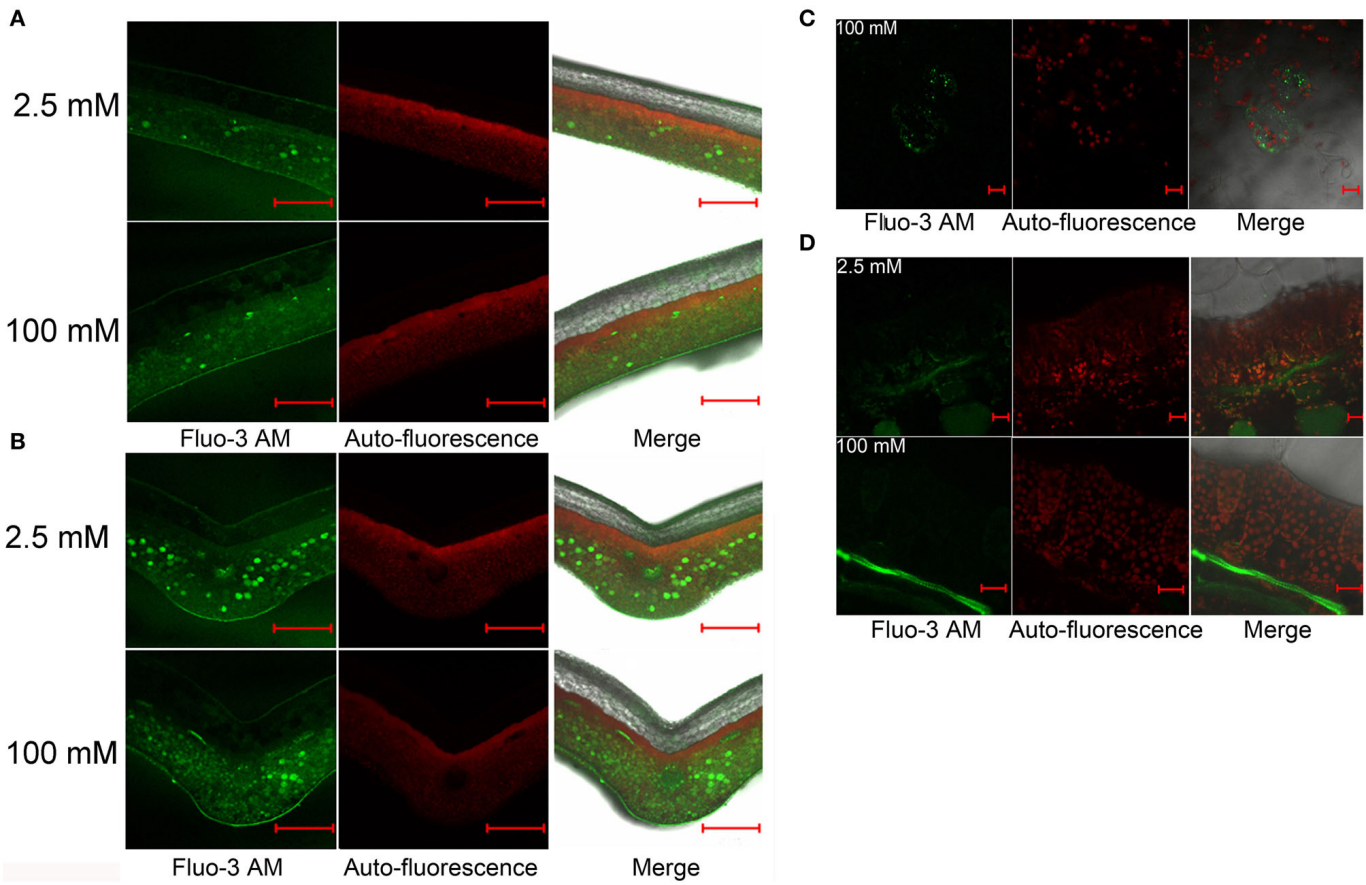
**Distribution of free Ca^2+^ in *L. pauciflorus* leaves detected by Fluo-3 AM staining after 2.5 and 100 mM Ca treatments for 7 days. (A,B)** Non-vein **(A)** and vein **(B)** parts of leaves. Scale bar = 1 mm. **(C)** Spongy mesophyll cells in 100 mM Ca–treated plants. Scale bar = 20 μm. **(D)** Palisade and spongy mesophylls. Scale bar = 20 μm.

Ca^2+^ is the active form of Ca in living cells with important regulatory roles. Several photosynthetic functions are influenced by Ca^2+^ (Brand and Becker, 1984; Long et al., 2005). As described above, we have demonstrated that *L. pauciflorus* and *B. hygrometrica* can survive and keep reasonable levels of photosynthesis under high Ca conditions, despite that large amount of Ca accumulated in leaves. To detect Ca^2+^ concentrations and distributions in leaves of *L. pauciflorus* and *B. hygrometrica*, Fluo-3 AM was used to stain leaf sections. The results showed that the major Ca signals were detected as faint Ca fluorescence in the large and transparent upper epithelial cells that showed no chlorophyll autofluorescence, and the narrow palisade cells that were rich of chlorophyll autofluorescence, while considerable Ca fluorescence in the elliptical spongy cells that showed relatively weak chlorophyll autofluorescence in plants under both 2.5 and 100 mM Ca treatment (Figures 6A,B). Particularly strong Ca fluorescence signals were found in some spongy cells that scattered randomly, which are more abundant near the vein. At high magnification, it is observed that the strong Fluo-3 AM signals was likely from vacuoles in many of these cells as the vacuole comprises the majority of the volume of these cells and the Fluo-3 AM staining is smeared throughout the entirety of many of these cells. The Fluo-3 signals were not overlapped with chlorophyll autofluorescence, indicating that Ca^2+^ was not accumulated to high level in chloroplasts under normal and high Ca treatment (Figures 6C,D).

**Figure 7:**
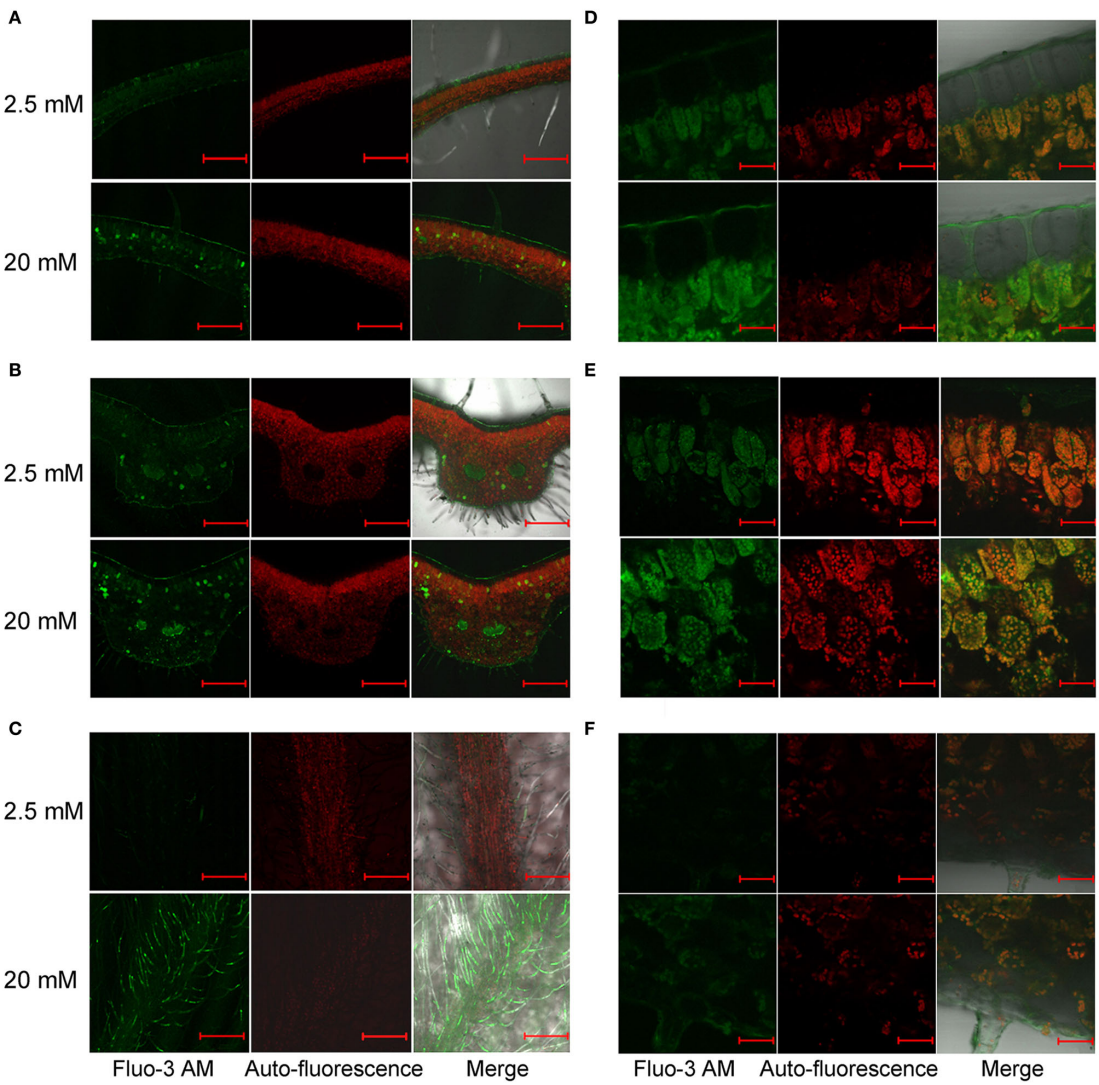
**Distribution of free Ca^2+^ in *B. hygrometrica* leaves detected by Fluo-3 AM staining after 2.5 and 20 mM Ca treatment for 7 days. (A,B)** Non-vein **(A)** and vein parts **(B)** of leaves. Scale bar = 1 mm. **(C)** Trichomes. Scale bar = 1 mm. **(D)** Upper epidermis. Scale bar = 50 μm. **(E)** Mesophyll cells. Scale bar = 50 μm. **(F)** Lower epidermis. Scale bar = 50 μm.

Fluo-3 AM staining showed a different pattern of Ca^2+^ distribution in leaves of *B. hygrometrica*. Weak fluorescence was detected in all cell types, and relatively stronger signals were found in vascular bundles and some cells that scattered randomly near the veins in 2.5 mM Ca-treated plants (Figures 7A,B). The number of these Ca-rich cells and the overall fluorescent intensity increased in 20 mM Ca-treated plants (Figures 7A,B). Besides, fluorescence was very strong in trichomes on the abaxial surface of 20 mM Ca-treated plants (Figure 7C). At high magnification, it was found that Fluo-3 AM signals in both palisade and spongy cells of 2.5 and 20 mM Ca-treated plants were smeared inside cells and mostly co-localized with the chlorophyll autofluorescence, indicating a large amount of Ca in chloroplasts (Figures 7D–F). Furthermore, nearly no Ca^2+^ signal was observed in upper epidermal cells but strong Ca^2+^ signals could be found in the cuticle layer outside the upper epidermis, which was spread from palisade cells via the apoplastic space (Figure 7D).

### Identification of Ca-inducible genes in *B. hygrometrica* that conferred Ca-resistant phenotype

**Figure 8:**
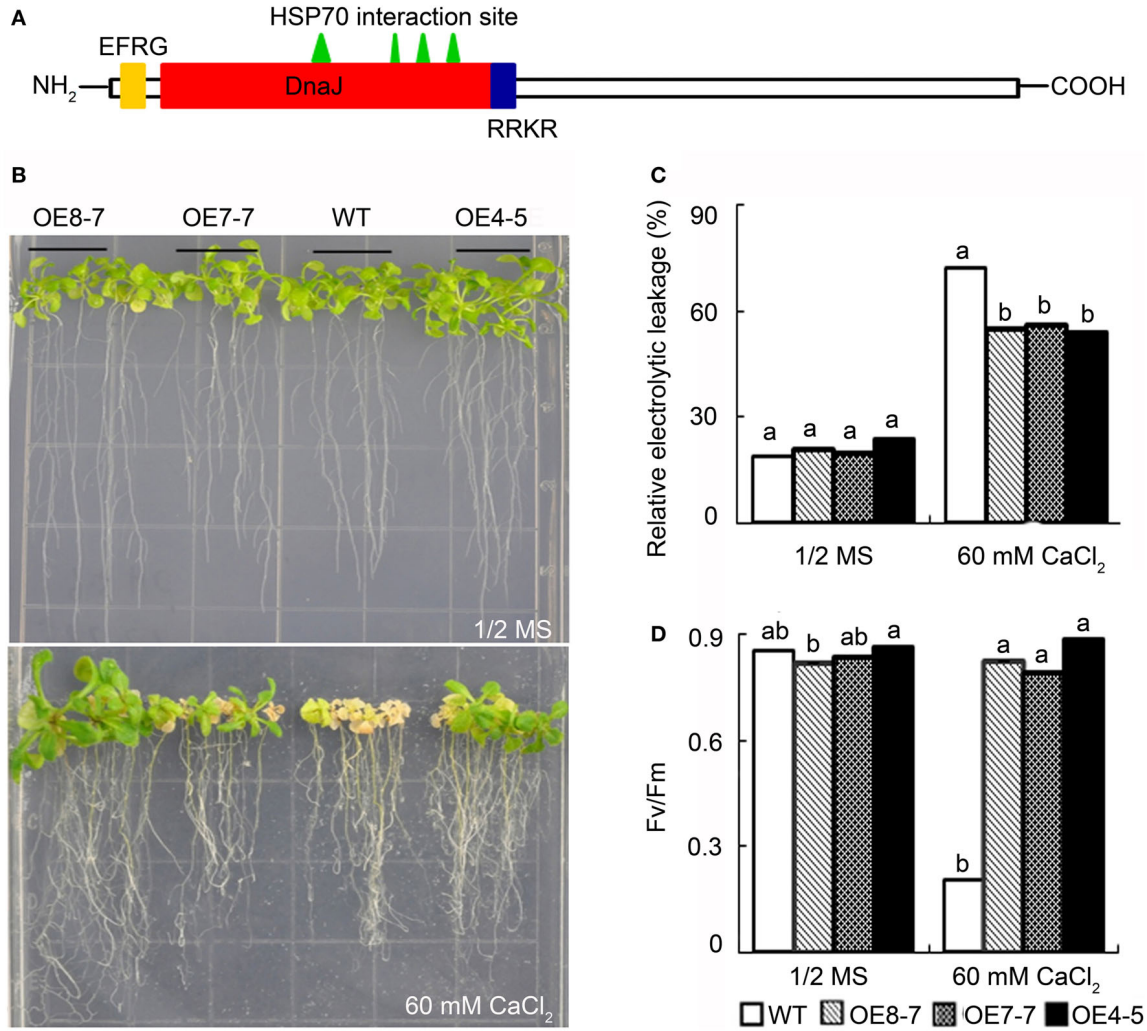
**Phenotype comparison of wild-type and transgenic plants overexpressing *BhDNAJC2* under high calcium treatment. (A)** Conserved domain of BhDNAJC2 protein. DnaJ, DnaJ domain; EFRG, endoplasmic reticulum membrane retention signal; RRKR, nuclear localization signal. **(B)** *Arabidopsis* seedlings grown on 1/2 MS and 1/2 MS+60 mmol·L^−1^ CaCl_2_ medium. Bar = 1 cm. **(C)** Relative electrolytic leakage. **(D)** Photochemical efficiency (Fv/Fm). Data presented as means ± SD (*n* = 6). WT, wild-type; OE8-7, OE7-7, OE4-5, transgenic lines overexpressing *BhDNAJC2*. The different lowercase letters on the bars indicate significantly different means (*P* < 0.05).

The observations described above have suggested that *L. pauciflorus* could prevent excessive Ca translocation to aboveground parts, leaves, palisade cells, and chloroplasts, thus limiting the harmful effects on photosynthesis and plant growth from high external soluble Ca stress. In contrast, *B. hygrometrica* could not avoid intracellular Ca accumulation in mesophyll cells even under low and moderate external soluble Ca environment, but could still keep active photosynthesis and normal growth. Given that such a trait is desired for the generation of Ca-rich crops/vegetables, it is meaningful to explore for genes with putative functions in Ca tolerance from *B. hygrometrica*. It was reported that Ca is preferentially stored in palisade and spongy cells in *Arabidopsis*, and *Arabidopsis* wild-type plants were severely impaired at >30 mM Ca application (Chan et al., 2003; Conn et al., 2011; Song et al., 2011). Thus, we used the previously generated transgenic *Arabidopsis* plants that overexpress *B. hygrometrica* genes to screen for Ca-resistant phenotype in 60 mM CaCl_2_ treatment. The tested genes included *BhC2DP1* (Zhang et al., 2012), *BhDNAJC2* (Chen et al., in press), and *BhOAR1* (Zhao et al., 2014). *BhC2DP1* encodes a plant-specific small protein with a single Ca^2+^-binding C2 domain, that had been reported to play a role in the crosstalk of ABA and Ca^2+^ signal pathway in response to drought stress (Zhang et al., 2012). *BhDNAJC2* encodes a DnaJ-domain containing heat shock protein (HSP) 40 member with endoplasmic reticulum membrane retention signal (EFRG) and nuclear localization signal (RRKR) (Figure 8A). It had been shown to localize in the endoplasmic reticulum (ER), nucleus, and cytoplasm, and play a key role in the resistance to osmotic, saline, alkaline, and heat stresses (Chen et al., in press). *BhOAR1* (*B. hygrometrica* Osmotic and Alkaline Resistance 1) encodes a 2-kb retro-element sequence that confers improved photochemical efficiency under both osmotic and alkaline stresses when transformed to *Arabidopsis* (Zhao et al., 2014).

As expected, the wild-type *Arabidopsis* grew normally on 1/2 MS plates, but became decolored, stunted and was dying when 60 mM CaCl_2_ was contained in the media. All the transgenic plants overexpressing *BhOAR1* and *BhC2DP1* were similar to wild-type under control and high Ca condition. Only plants overexpressing *BhDNAJC2* were able to confer tolerance to high Ca stress, as indicated by the obvious better growth on 1/2 MS plates containing 60 mM CaCl_2_, with bigger rosette, vivid and green leaves, and lower electrolytic leakage (Figures 8B–D). Moreover, the photochemical efficiency (Fv/Fm) was dramatically reduced in the wild-type plants under high Ca stress, but kept stable in the three lines of *BhDNAJC2* overexpression transgenic plants (Figure 8D), suggesting the efficient protection of photosynthesis-related proteins to maintain their function. In agreement with the previously characterized expression levels of *BhDNAJC2* in each transgenic line (Chen et al., in press), it was observed that OE8-7, the line with highest expression of *BhDNAJC2*, exhibited the strongest phenotypes. Together, these data indicates that the degree of tolerance of overexpression lines to high Ca is correlated to the overexpression of *BhDNAJC2*.

**Figure 9:**
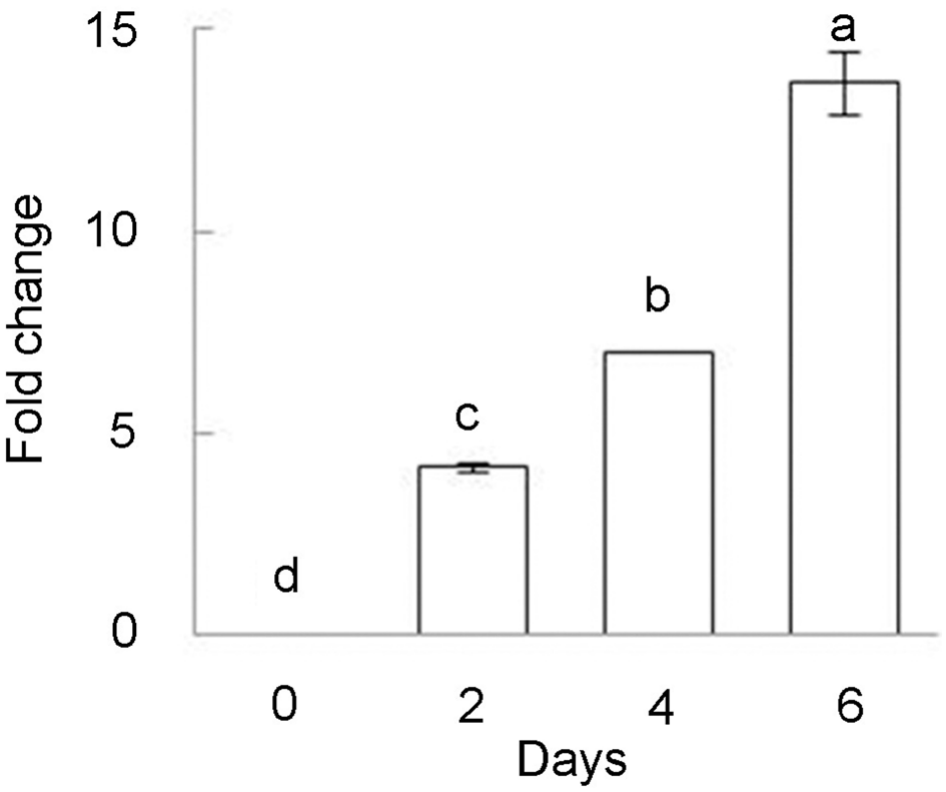
**Inducible expression of *BhDNAJC2* under various high Ca treatments in *B. hygrometrica***. Plants were treated with 20 mM CaCl_2_ for 2, 4, and 6 days. Mean values ± SD are shown (*n* = 3). The different lowercase letters on the bars indicate significantly different means (*P* < 0.05).

Previously only slight induction of *BhDNAJC2* expression was observed after Ca treatment for 2 h (Chen et al., in press). We further investigated the expression of this gene in *B. hygrometrica* under long-term high Ca stress. The results showed that *BhDNAJC2* was highly induced by Ca stress for 4 and 6 days (Figure 9). These results supported that *BhDNAJC2* may play an effective role in high Ca tolerance in *B. hygrometrica*.

## Discussion

Plants obtain Ca mainly from the soil solution (White, 2001). Soil in karst regions is rich in calcium, nearly three times more than in acid soils (Yuan, 2001; Cao et al., 2003). Not all plants are able to cope with such high external Ca in soil. For example, under laboratory experimental conditions, amaranth seedlings became stunted when external Ca was above 20 mM (Aguilar-Hernandez et al., 2011). *Arabidopsis* ecotype *Wassilewskija* (Ws) was severely impaired at 30 mM Ca application (Chan et al., 2003). In this study, *L. pauciflorus* seedlings were found to survive as high as 200 mM Ca without obvious growth defect or photosynthesis inactivation, demonstrating the extraordinary Ca tolerance of this species.

The root-to-shoot transportation of Ca in the xylem is driven by transpiration rate and soil Ca^2+^ concentration (Karley et al., 2000; Conn and Gilliham, 2010; Gilliham et al., 2011). In *L. pauciflorus*, the stomatal aperture and transpiration was obviously reduced with external Ca increase (Figure 2). A similar pattern was observed in *Arabidopsis* and *Commelina communis* (Silva et al., 1996; Tang et al., 2007). The quick response of stomatal closure may help to keep water content and normal physiological activities, thus representing a major factor for restricting Ca accumulation in *L. pauciflorus*. Besides, frequent drought stress is another major stress for plants inhabiting on the limestones in karst area. *L. pauciflorus* appears to have a much lower stomatal conductance and transpiration rate even under low Ca condition, suggesting that it is also suited to maintenance of water status. By contrast, in *B. hygrometica*, stomatal conductance, and transpiration were unchanged, which may account for the decrease of water content and photosynthesis under high Ca treatment. Previously it was observed that the stomata of *B. hygrometrica* were kept open under drought stress (Mitra et al., 2013). The unique regulation of stomata movement had been observed in many resurrection plants, while the molecular basis is still under investigation (Beckett et al., 2012; Gechev et al., 2012).

^45^Ca autoradiography, scintillation counting, SEM X-ray, and Fluo-3 AM staining had been used to study Ca sinks and distribution in different organs, tissues, and cell types in many plants (Zhang et al., 1998; Albrechtova et al., 2003; Peng et al., 2004; Busse and Palta, 2006; Fernandez-Garcia et al., 2009; Kerton et al., 2009). Using these techniques we were able to show that the major Ca sink(s) in stolon (Figure 3), and the preferential Ca storage in spongy cell compared to palisade cells in leaves (Figure 6) in *L. pauciflorus*. Stolon was also observed to be a Ca sink in *Oxalis acetosella* (Rodenkirchen, 1998). Giving that Ca preferentially moved to organs with high meristematic activity (Lotscher and Hay, 1996), and the meristems of expanding underground organs are known for their high Ca demand (Rodenkirchen, 1998), the growing stolon could greatly restrict Ca translocation to the aboveground organs. The movement of Ca from stem into leaves was further blocked under high Ca conditions by certain mechanisms including reduced stomatal aperture and transpiration. The ability of *L. pauciflorus* to prevent excessive leaf Ca accumulation may prevent a degree of physiological toxicity due to Ca stress in contrast to *B. hygrometrica*.

Palisade cells are the major sites for photosynthesis. Chloroplasts are unique organelles that are responsible for photosynthesis, but the increase in chloroplast Ca concentration will provoke the second ROS-burst, which is required to induce HR cell death in plants (Nomura et al., 2012). In these contexts, the preferential storage of Ca in spongy cells may help avoid Ca accumulation in palisade cells, thus contribute to the maintenance of active photosynthesis and cell vitality under high Ca environment. These observations, along with the observation that Fluo-3 AM detected free Ca^2+^ signals were not co-localized with chlorophyll auto-fluorescence (Figures 6C,D), have suggested that blocking Ca translocation to palisade cells and to chloroplasts may represent a second barrier to protect photosynthesis under high Ca condition. Cell-specific Ca^2+^ storage at tissue and cellular levels has been reported for many plants (Storey and Leigh, 2004; Kerton et al., 2009). It has been attributed to the different expression of particular Ca^2+^-transporters and Ca^2+^/H^+^-antiporters (Conn and Gilliham, 2010; Conn et al., 2011). For instance, *CAX1* was preferentially expressed in Ca-rich mesophyll, and its function in controlling mesophyll Ca concentration and preventing epidermal Ca accumulation also provide Ca stress tolerance and regulate gas exchange (Conn et al., 2011).

In contrast to the ability to survive high Ca in *L. pauciflorus, B. hygrometrica* can only survive moderate Ca stress (20 mM). However, the observations of higher Ca contents in leaves than that in roots (Figures 3C,D), the significant increase upon high Ca application (Figures 5, 7), the co-localization of Ca^2+^ signals with chlorophyll autofluorescence in both palisade and spongy cells (Figures 7D,E), and the consistent active photosynthesis (Figure 2) have indicated that *B. hygrometrica* has the ability to tolerate relatively high internal Ca. It is likely that there are certain mechanisms to minimize the harmful effects of excessive Ca and to protect photosynthetic apparatus in *B. hygrometrica*.

Abiotic stresses often interrupt protein folding, transportation, and stability, thus plastid protein homeostasis is critical during chloroplast biogenesis and responses to changes in environmental conditions. DnaJ proteins can interact with the ATPase domain of HSP70 and hydrolyse ATP to ADP, facilitating client capture (Kampinga and Craig, 2010), and function as molecular co-chaperones of HSP70 in protein folding, unfolding, and assembly as well as in cellular secretory pathways (Walsh et al., 2004). ER-localized heat-shock protein HSP90.7 has been reported to function in resistance to tunicamycin or high calcium-induced ER stresses (Chong et al., in press). In this study, we demonstrated that *BhDNAJC2* was significantly induced by long-term high Ca treatment (Figure 9), and the overexpression of *BhDNAJC2* in *Arabidopsis* could improve plant growth and photosynthesis under high Ca stress (Figure 8). Both chloroplast-targeted chaperones that protect photosystem II and enzymes in the carbon fixation reaction, and various factors that facilitate protein targeting to chloroplasts including HSP70 and HSP90 have been identified (Liu et al., 2005; Chen et al., 2010; Ajjawi et al., 2011; Lee et al., 2013; Kong et al., 2014). It is not known whether cytosolic DnaJ proteins such as BhDNAJC2 are involved in protein targeting processes. However, our previous and present observations that *Arabidopsis* plants overexpressing *BhDNAJC2* displayed better growth, higher Fv/Fm and lower electrolytic leakage under drought, salt, alkaline and heat stress conditions have suggested a general but effective role of BhDNAJC2 on safeguarding homeostasis and stabilization of proteins, including those related to photosynthesis under various environmental stresses (Chen et al., in press). However, a certain threshold concentration or expression level may limit the role of BhDNAJC2 in *B. hygrometrica*, for example, high expression of the gene in *Arabidopsis* can provide higher Ca tolerance than for *B. hygrometrica*. Giving that the HSP family is composed of a large number of members, and several other HSP genes had been reported to be induced by external Ca application (Zhang et al., 2013), further characterization of the interaction of BhDNAJC2 and other HSPs from *B. hygrometrica* will shine a light to understand the roles of chaperone-mediated protein quality control in the tolerance to high internal Ca in *B. hygrometrica*.

In summary, by comparing the ranges of high external soluble Ca that plants could survive, the accumulation and distribution patterns of Ca in different tissues and cells under various levels of external Ca, distinct strategies to cope with high Ca environment were sketched in two karst-habiting Gesneriaceae species *L. pauciflorus* and *B. hygrometica*. Further characterization of the tolerant mechanisms to high internal Ca in *B. hygrometrica* will provide resource genes for biogeneration of Ca-rich crops and vegetables, whereas the assessment of the molecular basis for controlling Ca levels in crucial tissues in *L. pauciflorus* will facilitate the biotechnological breeding of crops that could improve agriculture and ecology in karst regions.

### Conflict of interest statement

The authors declare that the research was conducted in the absence of any commercial or financial relationships that could be construed as a potential conflict of interest.

## Supplementary material

Figure S1 **Growth and physiological parameters of *L. pauciflorus* and *B. hygrometrica* plants treated with 20 (for *B. hygrometrica*) or 200 mM (for *L. pauciflorus*) Ca(NO_3_)_2_, CaCl_2_, and Mg(NO_3_)_2_ for 7 days, respectively. (A)** The growth of *L. pauciflorus*. Scale bar = 1 cm. **(B)** The growth of *B. hygrometrica*. Scale bar = 1 cm. **(C)** Net photosynthetic rates, relative water contents, osmolalities of leaves of *L. pauciflorus* and *B. hygrometrica* plants. The mean ± SD is shown (*n* = 3). The different lowercase letters on the bars indicate significantly different means (*P* < 0.05).
