# Supplementary figures and images for: A comparative study on Ca content and distribution in two Gesneriaceae species reveals distinctive mechanisms to cope with high rhizospheric soluble calcium
Source: Front Plant Sci. 2014 Nov 20;5:647. doi: 10.3389/fpls.2014.00647 (PMC4238373; doi:10.3389/fpls.2014.00647)

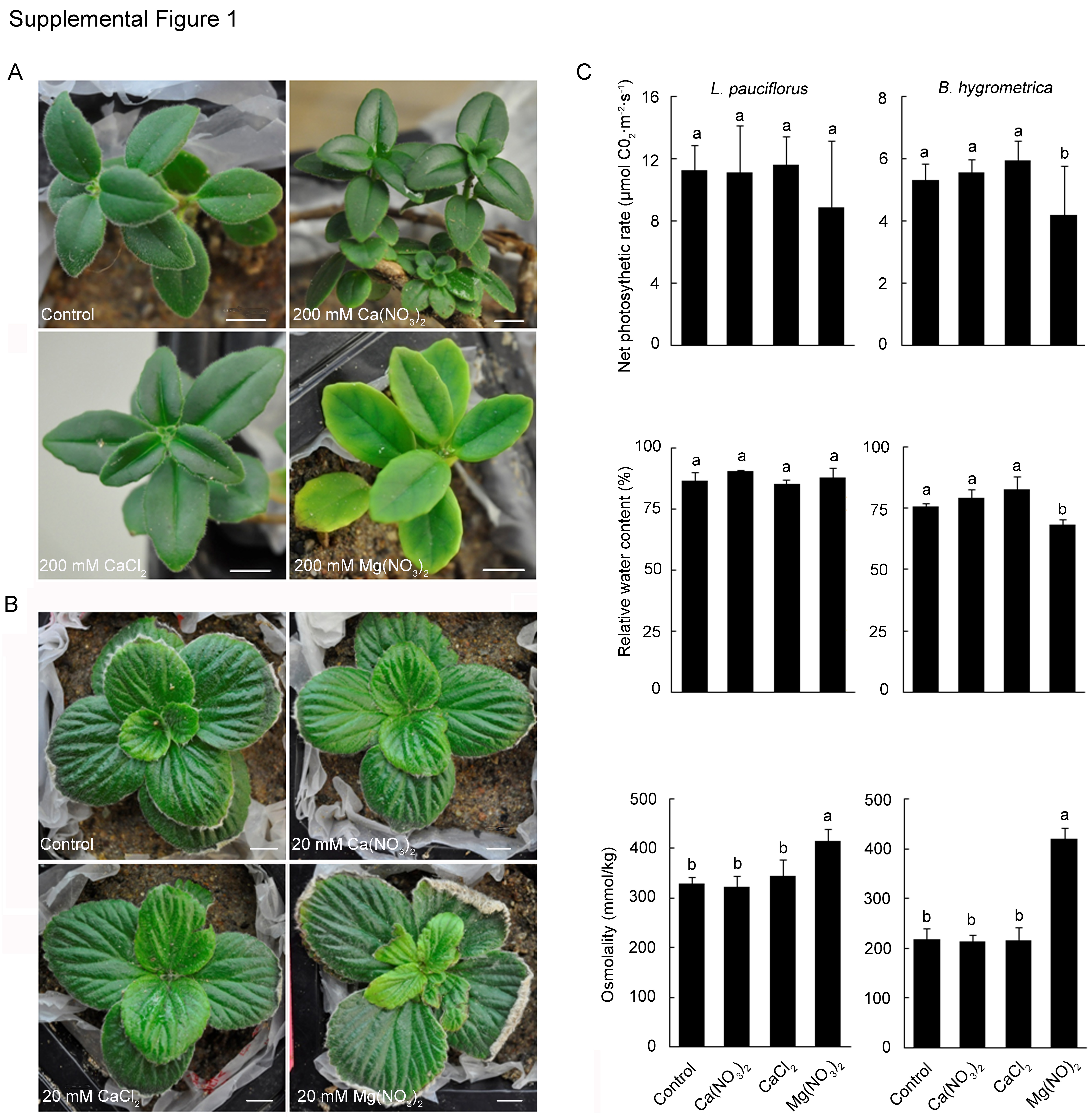

Supplement: Figure S1 — Growth and physiological parameters of L. pauciflorus and B. hygrometrica plants treated with 20 (for B. hygrometrica) or 200 mM (for L. pauciflorus) Ca(NO3)2, CaCl2, and Mg(NO3)2 for 7 days, respectively. (A) The growth of L. pauciflorus. Scale bar = 1 cm. (B) The growth of B. hygrometrica. Scale bar = 1 cm. (C) Net photosynthetic rates, relative water contents, osmolalities of leaves of L. pauciflorus and B. hygrometrica plants. The mean ± SD is shown (n = 3). The different lowercase letters on the bars indicate significantly different means (P < 0.05). [file Image1.JPEG]
